# A new hierarchical method for inter-patient heartbeat classification using random projections and RR intervals

**DOI:** 10.1186/1475-925X-13-90

**Published:** 2014-06-30

**Authors:** Huifang Huang, Jie Liu, Qiang Zhu, Ruiping Wang, Guangshu Hu

**Affiliations:** 1 Department of Biomedical Engineering, School of Computer and Information Technology, Beijing Jiaotong University, 3 Shang Yuan Cun, Hai Dian District, Beijing, China; 2 Department of Biomedical Engineering, School of Medicine, Tsinghua University, Beijing, China

**Keywords:** Heartbeat classification, Random projection, Support vector machine, Ensemble, Ventricular ectopic beat (VEB), Supraventricular ectopic beat (SVEB)

## Abstract

**Background:**

The inter-patient classification schema and the Association for the Advancement of Medical Instrumentation (AAMI) standards are important to the construction and evaluation of automated heartbeat classification systems. The majority of previously proposed methods that take the above two aspects into consideration use the same features and classification method to classify different classes of heartbeats. The performance of the classification system is often unsatisfactory with respect to the ventricular ectopic beat (VEB) and supraventricular ectopic beat (SVEB).

**Methods:**

Based on the different characteristics of VEB and SVEB, a novel hierarchical heartbeat classification system was constructed. This was done in order to improve the classification performance of these two classes of heartbeats by using different features and classification methods. First, random projection and support vector machine (SVM) ensemble were used to detect VEB. Then, the ratio of the RR interval was compared to a predetermined threshold to detect SVEB. The optimal parameters for the classification models were selected on the training set and used in the independent testing set to assess the final performance of the classification system. Meanwhile, the effect of different lead configurations on the classification results was evaluated.

**Results:**

Results showed that the performance of this classification system was notably superior to that of other methods. The VEB detection sensitivity was 93.9% with a positive predictive value of 90.9%, and the SVEB detection sensitivity was 91.1% with a positive predictive value of 42.2%. In addition, this classification process was relatively fast.

**Conclusions:**

A hierarchical heartbeat classification system was proposed based on the inter-patient data division to detect VEB and SVEB. It demonstrated better classification performance than existing methods. It can be regarded as a promising system for detecting VEB and SVEB of unknown patients in clinical practice.

## Background

An arrhythmia is a common and important symptom. It often occurs in patients with cardiovascular diseases. An arrhythmia is any abnormality in the rate, regularity, site of origin, or activation sequence of the electrical impulses of the heart. The type and frequency of arrhythmia can indicate the degree of danger. Broadly speaking, there are two types of arrhythmias. The first type seriously threatens the patient’s life and merits rescue treatment with a defibrillator. The other type is not life-threatening but still necessitates proper treatment to prevent serious problems. The present study targeted the second type of arrhythmia [1].

The electrocardiogram (ECG) is used to measure and record the electrical activities of the heart. The ECG is low-cost and non-invasive and has become a standard tool for diagnosing arrhythmias. Every beat of the heart produces a heartbeat. A typical ECG heartbeat waveform consists of a P wave, a QRS complex, and a T wave that indicate atrial depolarization, ventricular depolarization, and repolarization, respectively. Under normal circumstances, excitation from the sinoatrial node controls the rhythm of the heart. Any abnormality in normal sinus rhythm leads to an arrhythmia [2]. In ECG, many arrhythmias manifest as a series of heartbeats with abnormal intervals and waveform shapes. The rhythm of the ECG signal can be determined using classification of continuous heartbeats, and heartbeat classification is an important step in the identification of arrhythmias [1]. For accurate qualitative and quantitative analysis of heartbeats, thousands of heartbeats have to be recorded, and heartbeat classification is relatively time-consuming. Computers can be used for automated classification, and new algorithms must be developed to improve the performance of the classification process. Although heartbeat classification has been studied for decades, its performance on clinical data is unsatisfactory. Thus, researchers are continuing to develop methods of heartbeat classification.

In clinical applications, labeled ECG data are used to build a heartbeat classification system. Then this system is used to determine the types of heartbeats in unknown patients’ ECG recordings. The predictive ability of heartbeat classification systems largely depends on the method with which the dataset is partitioned during system construction. Currently, in most studies data partitioning is based on the heartbeat [3-10]. The subject to whom the heartbeat belongs (recording number) is not taken into account. Therefore, the training and testing datasets may come from the same patient, thus leading to optimistic classification results [11]. A heartbeat classification system based on such datasets may show poor predictive capability. This methodology is known as intra-patient heartbeat classification and is followed by a large number of heartbeat classification methods. Some training datasets include a global training set and local training sets. The global training set is independent of the testing set. Local training sets are from a section at the beginning of each test recording and are used to improve the classification performance on the rest of this recording. This technique is called patient-adaptive heartbeat classification [12-14]. With this method, because the local training sets and the testing set come from the same patient, the heartbeat classification is very accurate. However, it requires expert annotation and cannot be fully automated. In order to improve the predictive ability of automated heartbeat classification systems, the system should be constructed based on independent training and testing datasets (the training and testing sets come from different people). This is also called inter-patient classification. Inter-patient heartbeat classification is more difficult and more challenging than intra-patient classification and patient-adaptive classification, because it is difficult to detect similar heartbeats of the same class in both the testing and the training sets.

The Association for the Advancement of Medical Instrumentation (AAMI) has published standards for evaluating the performance of algorithms designed for detecting arrhythmias [15]. They recommend that different automatic arrhythmia analysis algorithms be assessed according to these standards. The AAMI divides the 15 types of heartbeats in the MIT-BIH arrhythmia database [16] into five classes including heartbeats originating in the sinus node (N), supraventricular ectopic beat (S or SVEB), ventricular ectopic beat (V or VEB), fusion heartbeat (F), and unknown beat type (Q). In particular, the AAMI is more concerned with the classification of SVEB (S) and VEB (V) than with that of other heartbeats classes. SVEB originates from the atria or from the atrioventricular node, and includes the four heartbeat types. Atrial premature beat (APB) is the most common heartbeat type and is caused by premature activation of the atrium prior to a normal heartbeat. Frequent APBs may indicate heart failure and atrial fibrillation, as well as other risk factors such as severe hypertension, asymptomatic atherosclerosis, or structural abnormalities resulting in a stroke, calcified mitral valve or a left atrial dilation. VEB mainly includes ventricular premature beat (VPB), which is the occurrence of an additional heartbeat from abnormal electrical activation of the ventricles prior to a normal heartbeat. Frequent VPBs may cause individuals with aortic stenosis, heart failure, or a history of heart attack or heart disease to have a higher risk of ventricular tachycardia and fibrillation, which can lead to sudden death [2,17]. Therefore, the detection of SVEBs and VEBs from a large quantity of class N beats is crucial.

In recent years, some researchers have used the AAMI standards to evaluate heartbeat classification methods based on the inter-patient classification schema [1,11,18-24]. de Chazal et al. [1] first proposed an automated heartbeat classification system using morphology and interval features, in which a weighted linear discriminant analysis (wLDA) classifier based on Bayesian decision theory was used. Llamedo et al. [11] and Mar et al. [19] both used the sequential floating forward selection algorithm and wLDA to select relevant characteristics from a variety of features, and improved the classification process in [1]. However, the training stage of this method was relatively time-consuming. In the heartbeat dataset, the distribution of different classes is imbalanced. When used with traditional classification methods, results become biased toward the majority class. de Lannoy et al. [20] adopted the feature sets selected by the wrapper method and the weighted support vector machine (wSVM) optimizing cost functions for heartbeat classification. This was found to further improve the SVEB sensitivity. Doquire et al. [21] applied a feature selection technique based on mutual information and the wSVM to heartbeat classification, but this did not improve the classification in [19]. According to the time dependence of waveform between heartbeats and the severe imbalances between classes, de Lannoy et al. [22] proposed a weighted conditional random field (wCRF) for heartbeat classification. This improved the average sensitivity, especially the SVEB sensitivity. All these methods are designed to identify different classes of heartbeats using the same features and same classifiers, but the resulting VEB and SVEB classification systems have not been found to be satisfactory. Zhang et al. [23] presented a disease-specific feature selection method to increase the performance of heartbeat classification and yet the sensitivities of SVEB and VEB were inferior to the results in [19]. Park et al. [24] proposed using hierarchical SVMs to solve the imbalance problem, but the class separability provided by the feature vector was slightly poor, resulting in an unsatisfactory performance.

**Figure 1:**
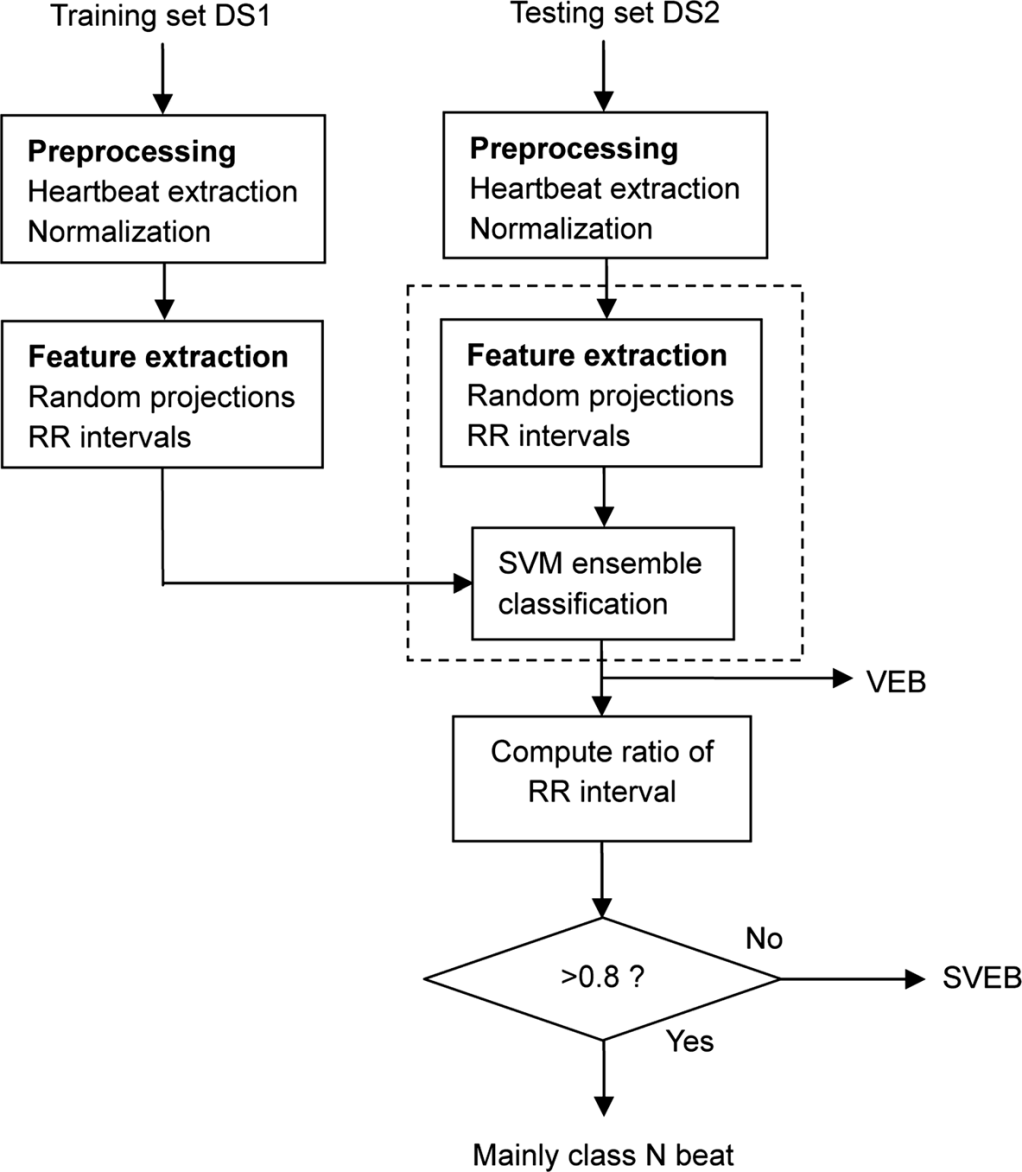
**Procedure to detect ventricular and supraventricular ectopic beat by random projections and RR intervals.** DS1 was used as the training set and DS2 as the testing set, both datasets based on inter-patient data partitioning. First, random projection and SVM ensemble were used to discriminate ventricular ectopic beat (VEB) from the preprocessed heartbeats, as shown in the dashed box (The details of this section will be introduced in Figure 2). Then the ratios of RR intervals were compared to a predetermined threshold of 0.8 to detect supraventricular ectopic beat (SVEB) from the remaining heartbeats.

In order to improve the VEB and SVEB detection performance, this study proposed a novel hierarchical heartbeat classification system in accordance with the AAMI guidelines (Figure 1). When the number of heartbeats is very high, due to the overlapping between various heartbeat classes, accurate heartbeat classification becomes difficult. In this study, according to the different characteristics of VEB and SVEB, we used different features and classification methods to detect VEB and SVEB. First we used random projection and SVM ensemble to detect VEB. To our knowledge, few studies have adopted random projection to extract the features of heartbeats. Random matrices are a very useful means of reducing dimensionality and compressing sensing [25,26]. Random matrices project the original high-dimensional data onto a low-dimensional subspace. It is computationally efficient and can effectively extract the waveform morphology features. Due to the randomness of the random matrix, feature extraction is unstable, but this characteristic was used to construct component classifiers and implement ensemble learning. This ensemble learning, using the diversity of component classifiers, can improve the generalization performance and avoid overfitting. SVM component classifiers were constructed on each group of random projections and RR intervals, and the class of heartbeat was determined using the maximum voting strategy combined with the output of multiple SVM classifiers. After VEBs were detected, for the remaining heartbeats, the ratio of the actual RR interval (the interval between successive R wave fiducial points) to the mean RR interval was compared to a predetermined threshold to detect SVEB. Use of the RR interval ratio can reduce the overlap between SVEB and class N heartbeat and thus increase the SVEB detection rate. The optimal parameters for SVMs were selected using cross-validation and the threshold was determined according to the sensitivity and positive predictive value of SVEB on the training set. We also assessed the effect of different lead configurations on the classification results of VEB. The experimental results showed that the method with configuration lead A classified heartbeats better than other configuration methods did.

## Methods

### ECG data

Datasets used in the present study were obtained from the MIT-BIH arrhythmia database (http://www.physionet.org/physiobank/database/mitdb/) [16]. These included forty-eight 30 minute ECG recordings collected from two leads (here labeled as lead A and B). There are a total of 109,492 heartbeats, from 15 heartbeat types. Each of the five heartbeat classes defined by the AAMI (N, S, V, F, and Q) includes one or more types of heartbeats. The correspondence between these five classes and the 15 heartbeats types in the MIT-BIH arrhythmia database can be found in the reference [1]. As in other methods [11,19-24], recordings in the MIT-BIH arrhythmia database were divided as described in [1], to facilitate the comparison of different methods. Consistent with the standard recommended by the AAMI, four heartbeat recordings in which a paced beat was involved were discarded [1]. The remaining 44 recordings were divided into two datasets, DS1 and DS2, each containing 22 recordings. DS1 and DS2 contained similar types of heartbeats, about 50,000 heartbeats in each dataset, and they included both simple and complex arrhythmia recordings. DS1 was used as the training set and DS2 as the testing set. The numbers of different heartbeat classes under the AAMI standard in the two datasets are shown in Table 1. The training set DS1 included 45868 class N beats, 942 SVEBs, 3787 VEBs, 415 class F beats, and 8 class Q beats. The testing set DS2 included 44258 class N beats, 1837 SVEBs, 3221 VEBs, 388 class F beats, and 7 class Q beats.

**Table 1:** Distribution of AAMI heartbeat classes in the two independent datasets

**Dataset**	**N**	**S (SVEB)**	**V (VEB)**	**F**	**Q**	**Total**
DS1	45868	942	3787	415	8	51020
DS2	44258	1837	3221	388	7	49711

The MIT-BIH arrhythmia database was divided into two datasets as described in [1]. N, S (SVEB), V (VEB), F and Q refer to heartbeats originating in the sinus node, supraventricular ectopic beat, ventricular ectopic beat, fusion heartbeat, and unknown beat type, respectively. Four recordings (102, 104, 107 and 217) containing paced beats were not included. Dataset DS1 includes data from recording 101, 106, 108, 109, 112, 114, 115, 116, 118, 119, 122, 124, 201, 203, 205, 207, 208, 209, 215, 220, 223 and 220. Dataset DS2 includes data from recording 100, 103, 105, 111, 105, 7, 11, 121, 123, 200, 202, 210, 212, 213, 214, 219, 221, 222, 228, 231, 233 and 234.

### Overview of the proposed method

The heartbeat classification process consists of three parts: preprocessing, VEB detection, and SVEB detection. Figure 1 shows the stages of the proposed system for heartbeat classification in this study.

Firstly, the preprocessing stage extracted the heartbeats from ECG signals using the R peak times provided by the MIT-BIH arrhythmia database and then normalized the heartbeats to reduce the possible incorrect decisions.

Afterwards, we employed random projections of the preprocessed heartbeats together with RR intervals and SVM ensemble to detect VEB. Due to the randomness of the random matrices, multiple random matrices can generate multiple groups of random projections of the preprocessed heartbeats and thus multiple SVM classifiers were trained on the training set DS1 accordingly. The class of heartbeat was determined using the ensemble of multiple SVM classifiers.

Finally, after VEBs were detected, the ratio of the RR interval for the remaining heartbeats was computed and compared to a predetermined threshold to detect SVEB. In what follows, we detailed the three main stages in our study.

### Preprocessing

Heartbeat extraction requires detection of the QRS complex. The main concern of the present study was heartbeat classification. Therefore, the detection of the QRS complex was not explored. Instead, reference points of the R wave provided by the annotation files in the MIT-BIH arrhythmia database were used directly. The ECG sampling frequency was 360 Hz. Each heartbeat sample included 200 data points, all collected from 0.278 seconds (s) before to 0.278 s after the R wave. In this way it contained a complete heartbeat signal, including the QRS complex, P wave, and T wave. To reduce wrong decisions caused by equipment error and individual variations, each heartbeat sample was subtracted by the mean and then divided by the standard deviation. The resultant normalized waveform was treated as the preprocessed heartbeat. No denoising of the ECG signal was performed, mainly because denoising with filters may remove certain details from the heartbeat waveform and affect subsequent classification.

### VEB detection

Feature extraction is crucial to pattern classification. The main characteristics of VEB are that it has a wide QRS complex and the RR intervals are shorter than class N and F beats. Therefore, it is reasonable to choose the morphological features and RR intervals as the features of heartbeats. However, the morphological features of the preprocessed heartbeats are not employed directly due to the diverging in the feature space and high dimensionality. Instead, the transform methods are often used to prevent feature vectors from diverging in the feature space. Random projection is an extremely useful tool for reducing dimensionality and for compressing sensing. Compared to other feature extraction methods such as entropies, higher order statistics, as well as other non-linear methods, random projection can retain enough information with low dimension to recover the original signal [25,26]. The information is non-redundant and is vital in identifying different heartbeat classes. In addition, random projection can be generated with high efficiency without the need for a special algorithm. Therefore, random projection has hope to become a useful feature extraction method. As far as we know, few studies have utilized random projection as a feature extraction method for heartbeat classification. In this study, we employed random projection to extract the morphological features of the heartbeats. Due to the randomness of the random matrix, feature extraction is unstable, but we used SVM ensemble to overcome this problem. On the other hand, ensemble learning can improve the generalization performance and avoid overfitting by enhancing the diversity of the component classifiers.

**Figure 2:**
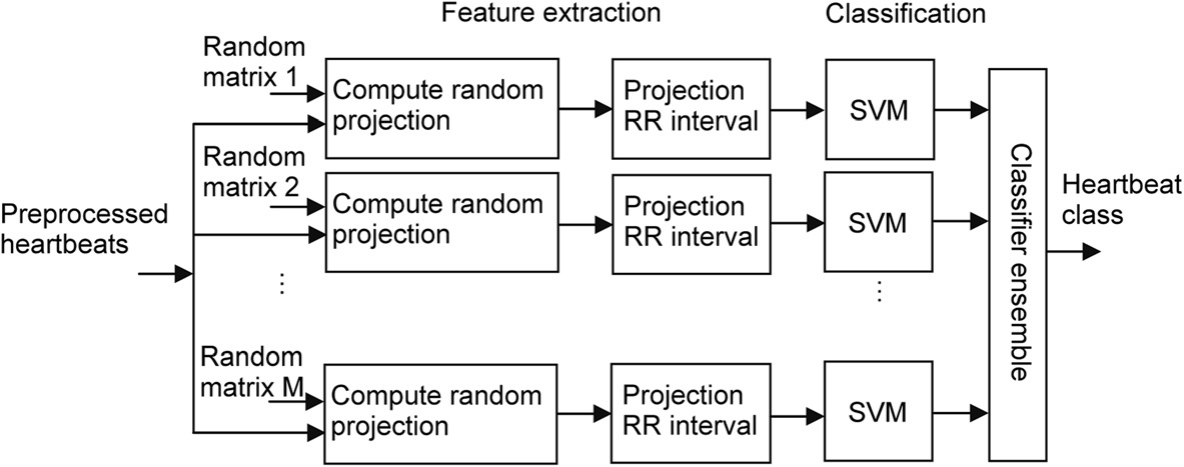
**Schematic representation of detecting ventricular ectopic beat (VEB).** M random matrices were generated and the preprocessed heartbeats were projected onto them to compute random projections. SVM component classifiers were constructed based on each group of random projections and RR intervals. The type of heartbeat was determined using majority voting strategy to combine multiple SVM classifiers.

VEB classification is illustrated in Figure 2. First, *M* random matrices were generated, and the preprocessed heartbeat was projected onto the random matrices to calculate the *M* groups of random projections. Feature vectors were composed of random projections and RR intervals. One SVM component classifier was constructed per group of random projections and RR intervals using the training dataset. Then the classes of heartbeats of the testing dataset were obtained using the integrated results of these *M* SVM classifiers.

#### Extraction of heartbeat features using random projection

Random matrices are often used to reduce dimensionality [25]. Recently, they are applied as a sensing matrix to measure original signals in compressive sensing [26]. Using a random matrix to extract features is equivalent to using a sensing matrix to measure original signals, i.e., projecting the original signals onto a random matrix to produce random projections.

Assuming that in random matrix ***A*** ∈ *R*^*d* × *m*^, the rows are the *m* dimensions of independently sampled random signals, *d* being the number of random signals. Data matrix ***X*** ∈ *R*^*n* × *m*^ is the original set of signals containing *n* groups of *m*-dimensional heartbeat samples. Given known matrices ***A*** and ***X***, random feature matrix ***F*** ∈ *R*^*n* × *d*^ can be expressed as follows:

(1) $$F=X{\left(A^{+}\right)}^{-1}$$

here (***A***^+^)^- 1^is the pseudo-inverse of random matrix ***A***; ***F*** is a feature set consisting of *n d*-dimensional random feature vectors. This operation projects the original *m*-dimensional heartbeat samples onto the *d*-dimensional feature space (*d* < *m*) using a *d* × *m* random matrix. The projection coefficients in each row of matrix ***F*** are the random features extracted from the heartbeat. The operation of random projection is very simple to implement via matrix operations and does not require any special algorithm.

**Figure 3:**
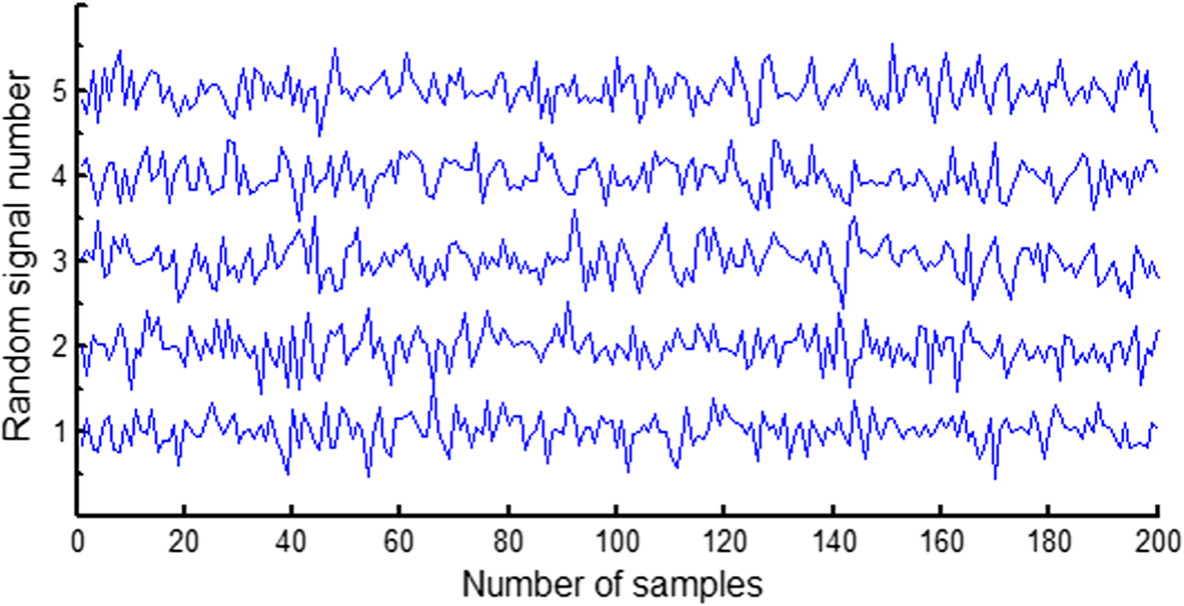
**Gaussian random signals for forming a random matrix.** The 50 random signals composed a 50 × 200 random matrix whose row is a random signal. Five random signals are shown and the vertical axis represents the random signal number.

**Figure 4:**
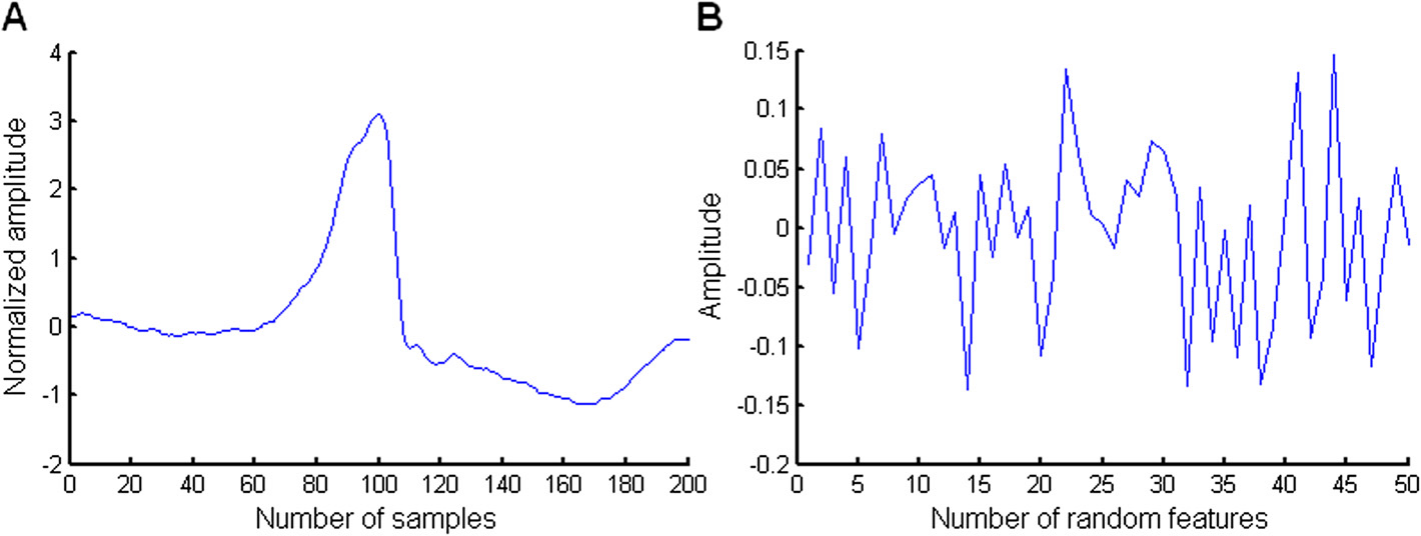
**A preprocessed heartbeat and its random projection. A**: A preprocessed premature ventricular contraction. **B**: The random projection of the premature ventricular contraction shown in **A**.

In order to obtain a satisfactory heartbeat classification performance, during feature extraction using random matrices, the composition of the random matrices, the number of random signals and the number of random matrices need to be taken into consideration. Experiments conducted by Bingham et al. have shown that Gaussian random matrices are rather effective in dimension reduction [25]. For this reason, in the present study, Gaussian random matrices were used. The number of random signals determines the dimension of the random projection. A low projection dimension is conducive to increasing the classification speed, but if the projection dimension is too low, the classification performance will be reduced. The number of random matrices determines the number of classifiers, and when choosing the optimal number of random matrices, both classification speed and classification performance must also be taken into account. In fact, after the number of random matrices reaches a certain value, further increases will no longer improve the classification performance, but rather reduce the classification speed. From the results of preliminary experiments, the number of random signals and the number of random matrices were set at 50 and 15, respectively, to achieve satisfactory classification at a reasonable speed.We generated fifty 200-dimensional Gaussian random signals with a mean of 0 and a standard deviation of 1 using a MATLAB function and five random signals, as shown in Figure 3. The 50 random signals composed a 50 × 200 random matrix where the row is a random signal. A total of 15 Gaussian random matrices were generated. The heartbeat samples were separately projected to onto fifteen 50 × 200 Gaussian random matrices according to Equation (1). As a result, 15 groups of 50-dimensional random projections for single lead data were generated. Figure 4 (A) illustrates a preprocessed heartbeat sample with premature ventricular contraction, and Figure 4 (B) shows a random projection of the premature ventricular contraction illustrated in Figure 4 (A). The RR interval is a very important feature for describing many ECG arrhythmias. The RR interval here refers to the interval between the present heartbeat and the previous heartbeat. The feature vector of the heartbeat was composed of a 50-dimensional random projection and the RR interval. A total of 15 different 51-dimensional feature vectors were generated for each heartbeat. Therefore, the training set DS1 and the testing set DS2 have 15 separate groups of feature vectors separately, where each group was generated by the same random matrix.

#### Construction of SVM ensemble classifiers

SVM is a widely applied classification method based on structural risk minimization. In SVM, the original low-dimensional space is mapped into the high-dimensional feature space through a kernel function, and then the optimal separating surface in the high-dimensional feature space is found to maximize the margin between the training data and the decision boundary [27]. Compared to other classifiers, SVM can obtain better classification performance through the selection of the kernel function and model parameters, and it shows good generalization performance. SVM is simple to perform with a fast and efficient software package developed by Chang et al. [28]. SVM has become one of the most popular classifiers at present, and thus, it was used to detect VEB in this study.

The SVM ensemble classifiers were constructed based on the five classes of heartbeat feature vectors of the training set DS1. This training set had 15 groups of training feature vectors. We constructed one SVM on each group of training feature vectors and thus generated 15 SVMs. Each SVM was then used to classify the corresponding group of testing feature vectors (generated with the same random matrix as the training feature vectors used). Fifteen SVMs suggested 15 heartbeat class labels for each test heartbeat. The class labels originated from classes N, SVEB, VEB, Q, and F. With a maximum voting strategy, the votes for the five classes were counted. The test heartbeat was then assigned to the class label with the highest number of votes.

The performance of SVM ensemble classification depends largely on the kernel function and model parameters. In the present study, the 15 SVMs all used the same kernel function and model parameters. The radial basis function (RBF) was used as the kernel function; the penalty parameter *C* and the kernel parameter *δ* were determined with cross-validation experiments to obtain the optimal classification performance. The SVM experiments were performed in MATLAB using libsvm-mat-2.91, which adopted a one-to-one method to achieve multi-group SVM [28].

#### The choice of SVM parameters using cross-validation

The penalty parameter *C* and the kernel parameter *δ* of 15 SVMs were set at the same values, which were determined with leave-one-out cross-validation on the training set DS1. Cross-validation is a model validation technique to evaluate the accuracy of a predictive model performance in practice. For each cross-validation, a dataset is determined to test the classification model on the training set to avoid overfitting. The training set DS1 included 22 recordings. In cross-validation, one recording was used as a testing set. The remaining 21 recordings served as the training set. One classification confusion matrix for each cross-validation was generated using the described SVM ensemble classifiers. The cross-validation process was repeated 22 times, and 22 classification confusion matrices were generated. Then the 22 classification confusion matrices were added, and the sensitivity and positive predictive value for class N heartbeat and for VEB were calculated. The advantage of this processing method is that it can be used to avoid errors caused by averaging, and thus the result is a faithful reflection of the actual classification.

**Figure 5:**
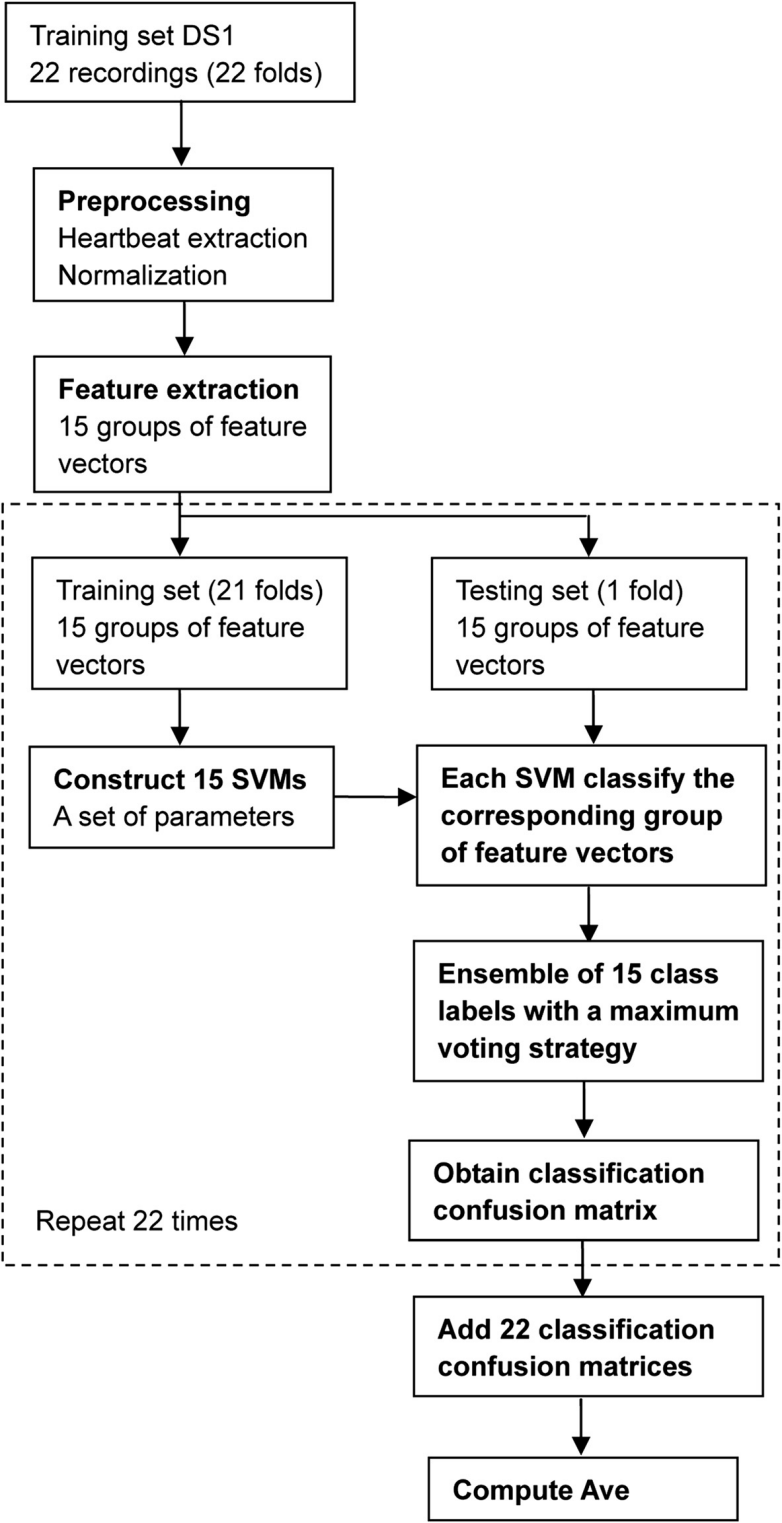
**The cross-validation process for the parameter choice in VEB detection on training set DS1.** The part in the dashed box is required to repeat 22 times.

The following three values were tested for the penalty parameter *C*: 1, 10, and 100. The following four values were tested for the kernel parameter *δ*: 0.4, 0.7, 1.0, and 1.3. The optimal parameters were selected from a total of 12 combinations, and evaluated using the average value of sensitivity and positive predictive value of class N heartbeat and VEB (denoted as Ave). The larger the value of Ave was, the better the detection performance of VEB was. The group of parameters with the largest value of Ave was selected as the optimal parameter group.Based on the assumption that we finished the feature extraction of heartbeats and the training set DS1 had 15 groups of feature vectors, the entire process of the parameter choice is summarized as follows and shown in Figure 5:

**Step 1:** For each cross-validation, one recording was used as a testing set, composed of 15 groups of heartbeat feature vectors. Fifteen groups of heartbeat feature vectors taken from the remaining 21 recordings composed the training set.

**Step 2:** Each SVM with RBF kernel was constructed based on a group of training feature vectors with a set of parameters, and then used to classify the corresponding group of testing feature vectors. This process was repeated 15 times because of the existence of 15 groups of training and testing feature vectors. Fifteen SVMs suggested 15 heartbeat class labels for each test heartbeat. With a maximum voting strategy, the votes for the five classes, including N, SVEB, VEB, F, and Q, were counted. The test heartbeat was then assigned to the class label with the highest number of votes. The classification confusion matrix was stored.

**Step 3:** The cross-validation process in steps 1 and 2 was repeated 22 times (as shown in the dashed box in Figure 5). For each repetition, the testing set recording was changed. The 22 classification confusion matrices were added. The average value of sensitivity and positive predictive value of class N heartbeat and VEB (denoted as Ave) was computed.

**Step 4:** The parameters were changed and another set of parameters was utilized from 12 combinations (the penalty parameter *C*: 1, 10, and 100; the kernel parameter *δ*: 0.4, 0.7, 1.0, and 1.3). The 22-fold cross-validation process in steps 1, 2, and 3 was repeated 12 times. The group of parameters with the largest value of Ave was selected as the optimal parameter group.

**Step 5:** With the optimal parameters, 15 SVMs were constructed based on 15 groups of feature vectors in the training set DS1. These could be utilized for the subsequent classification.

#### The lead selection

ECG data included data collected from two leads, lead A and lead B, and the experiments were performed to assess the effect of lead configuration on the classification results. For two-lead data, the random projections of data collected from lead A were concatenated with those of data collected from lead B. Together with the RR interval, a 101-dimensional feature vector was obtained. Under the optimum parameters, three classification models with different lead configurations were compared, and the one exhibiting the best VEB detection performance on the training set DS1 was used to predict the testing set DS2.

### SVEB detection

**Figure 6:**
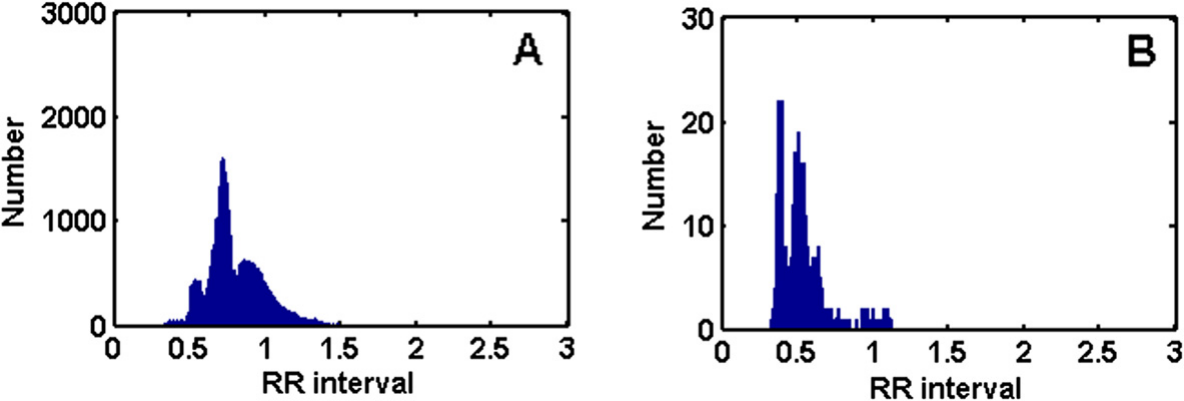
**Histograms of class N beat and SVEB RR intervals in the training set DS1. A**: The histogram of class N beat RR interval. **B**: The histogram of SVEB RR interval.

**Figure 7:**
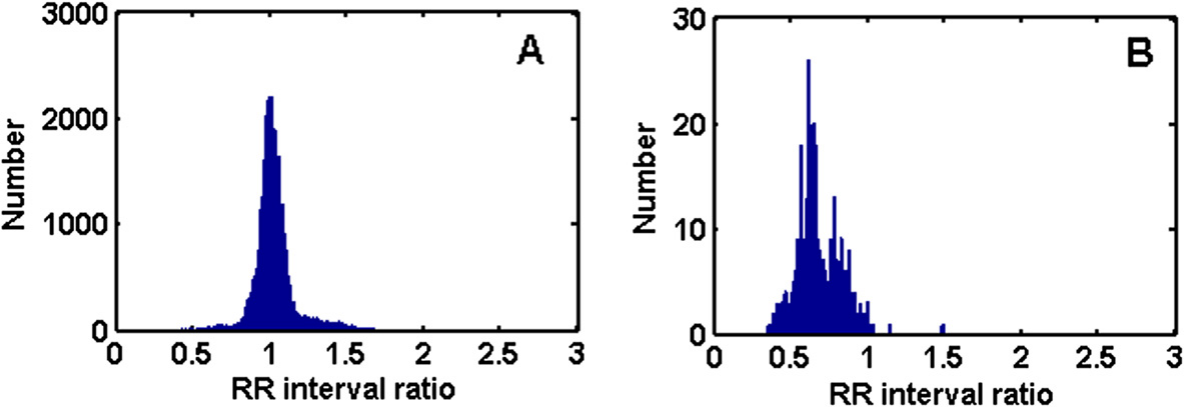
**Histograms of class N beat and SVEB RR interval ratios in the training set DS1. A**: The histogram of class N beat RR interval ratio. **B**: The histogram of SVEB RR interval ratio.

After detection of VEBs, the largest number of remaining heartbeats belongs to class N beats, followed by SVEBs. Because the waveform of SVEB is similar to that of class N heartbeat, the detection of SVEBs using waveform information is difficult. Therefore, we used the RR interval to detect SVEB. Histograms of class N heartbeat and SVEB RR intervals in the training set DS1 are shown in Figure 6. The RR intervals of SVEBs tended to be smaller than those of class N heartbeats, but the two sets of RR intervals overlapped across a relatively large range. Hence, it would be difficult to detect SVEB with direct use of the RR interval.The RR intervals of the majority of class N heartbeats are relatively similar to those of the normal beats. There are also many class N heartbeats with short RR intervals. This is caused by fast heartbeats, and such condition typically persists in the same recording. Therefore, the ratio of the RR interval to the mean RR interval of the entire recording was used to detect SVEB. Histograms of class N heartbeat and SVEB RR interval ratios in the training set DS1 are shown in Figure 7. When the RR interval ratio was used, the distribution of class N heartbeat changed. There were much fewer class N heartbeats with low RR interval ratios than before. Therefore, selecting an appropriate threshold was found to notably enhance the SVEB detection performance. In the testing set DS2, the RR interval ratio of each heartbeat was compared to the selected threshold. If the RR interval ratio was smaller than the threshold, that heartbeat was considered an SVEB. In this way, no classifier was needed, and the complexity of the algorithm was reduced and the detection rendered more efficient.

Similarly, to determine the threshold value for the detection of SVEB, the tested threshold values were set to 0.7–0.9, with a step size of 0.01. The sensitivity and positive predictive value for SVEB at different thresholds were compared to obtain the optimal threshold. Classification model selection was completed on the training set DS1, and the final classification performance was evaluated on the testing set DS2. Because DS2 was not involved in any previous calculations, the results can be considered a fair assessment of the classification model with respect to predicting unknown data.

### Classification of the test heartbeats using the proposed hierarchical method

Following the construction of the SVM ensemble classifiers and the threshold selection of an RR interval ratio based on the training set, the heartbeats in the testing set DS2 were classified using the proposed hierarchical method. The entire classification process is summarized in the following two sections:1) VEB detection (as shown in Figure 2): The preprocessed test heartbeats from a single lead were projected onto fifteen 50 × 200 random matrices (separately generated using fifty 200-dimensional Gaussian random signals) to produce fifteen 50-dimensional random projections. For two-lead data, random projections of data collected from lead A were concatenated with those of data collected from lead B. Fifteen random projections of the test heartbeat, together with the RR interval, formed 15 groups of test feature vectors. These groups were used as the input feature vectors of the 15 corresponding SVMs respectively. All SVMs utilized the same optimal parameters that were determined with cross-validation experiments on the training set. Fifteen SVMs suggested 15 heartbeat class labels for each test heartbeat. There were a total of five class labels, which included N, SVEB, VEB, F, and Q. With a maximum voting strategy, the votes for the five class labels were counted. The test heartbeats were then assigned to the class labels with the highest number of votes. We selected the test heartbeats that were classified to be VEBs.

2) SVEB detection: The heartbeats classified as VEB were removed from a total of 49,711 test heartbeats. We detected the SVEBs from the remaining test heartbeats. The RR interval ratios of the remaining heartbeats were computed and compared with the threshold determined on the training set. If the RR interval ratios were smaller than the threshold, the test heartbeats were classified as SVEBs.

### Experimental setup

To assess the performance of the heartbeat classification process, the sensitivity (Se), positive predictive value (PP), and accuracy (Acc) of the classification system were calculated. The sensitivity here refers to the relative number of correct detections of a particular class of heartbeats. The positive predictive value refers to the relative number of true positive heartbeats in all detected heartbeats of that class. The accuracy refers to the relative number of correctly detected heartbeats in all heartbeats. All these performance indicators can be calculated using the classification confusion matrix.

We carried out several experiments to construct and evaluate the proposed method. First, the classification models of VEB were constructed on the training set DS1. The same optimum values of the penalty parameter *C* and the kernel parameter *δ* for 15 SVMs were determined with leave-one-out cross-validation. The cross-validation experiments were performed to assess the effect of different lead configurations on the classification results of VEB based on the training set. For each lead configuration, the optimum parameters were identified. The effect of different lead configurations on classification results of VEB in the testing set DS2 was evaluated with the optimum parameters. Second, according to sensitivity and positive predictive value of SVEB in the training set DS1, the detection threshold of SVEB was determined. The results of SVEB were computed with the optimum threshold. Finally, we presented the classification confusion matrix and the classification results on each recording for SVEB and VEB detection.

Since heartbeat classification was expected to perform fast, the time of detecting VEB and SVEB was accessed on the testing set DS2. Computer configurations were as follows: CPU Intel Core Duo, clock rate 3 GHz, memory 3.25 GB, Windows XP operating system; the programs were written and run under MATLAB2007a.

## Results

In this study, we expected to obtain better classification performance via a novel hierarchical heartbeat classification system. According to different characteristics of VEB and SVEB, we first used random projection and SVM ensemble to detect VEB and then compared the ratio of the RR interval with a predetermined threshold to detect SVEB. We will present the results of several experiments to evaluate the proposed method.

### VEB cross-validation results of different parameters and different lead configurations on the training set

The cross-validation results of SVM ensemble classification under different parameters and lead configurations are listed in Table 2. For each lead configuration, the highest value of Ave is represented in bold and the corresponding parameter values are optimal. The cross-validation results of different lead configurations on the training set DS1 at these optimal parameter values are shown in Table 3. The detection results of class N beats are also shown because the optimal SVM parameter values were chosen based on the average value of sensitivity and positive predictive value of class N heartbeat and VEB (Ave). The results show that of the three configurations, when only lead A was used, the Ave was the highest. Meanwhile, at configuration lead A, the detection performance of VEB was the best with leave-one-out cross-validation. The sensitivity was the highest at 83.1% and the positive predictive value was slightly low at 57%. A number of class N beats and SVEBs were misclassified as VEBs. This indicates that the Ave can be utilized to assess classification performance effectively. Therefore, through the training process, we obtained the optimal parameter values under different lead configurations. The parameter values would be used for the VEB detection on the testing set DS2.

**Table 2:** VEB cross-validation results under different parameters and lead configurations on training set DS1

**Parameters**		**Ave (%)**		
***δ***	***C***	**Lead A**	**Lead B**	**Lead A + B**
0.4	1	81.4	**80.2**	79.2
0.4	10	83.0	71.3	81.8
0.4	100	80.9	67.8	80.8
0.7	1	82.5	74.8	81.1
0.7	10	82.3	69.6	81.5
0.7	100	80.9	65.8	81.1
1	1	83.0	73.3	81.9
1	10	81.6	68.8	81.0
1	100	81.3	65.2	81.2
1.3	1	**83.2**	73.2	**82.0**
1.3	10	81.4	68.4	80.9
1.3	100	81.7	64.8	81.3

Ave is the average value of sensitivity and positive predictive value of class N heartbeat and VEB. For each lead configuration, the highest value of Ave is represented in bold and the corresponding parameter values are optimal.

**Table 3:** VEB cross-validation result comparison of different lead configurations under optimal parameters on training set DS1

**Method**	**Parameters**		**N (%)**		**V (VEB) (%)**		**Acc (%)**	**Ave (%)**
	***δ***	***C***	**Se**	**PP**	**Se**	**PP**		
Lead A	1.3	1	95.4	97.4	83.1	57.0	92.5	83.2
Lead B	0.4	1	98.2	95.4	58.6	68.5	92.7	80.2
Lead A + B	1.3	1	92.6	97.1	80.3	57.9	89.9	82.0

Ave is the average value of sensitivity and positive predictive value of class N heartbeat and VEB.

### VEB detection results of different lead configurations on the testing set

**Figure 8:**
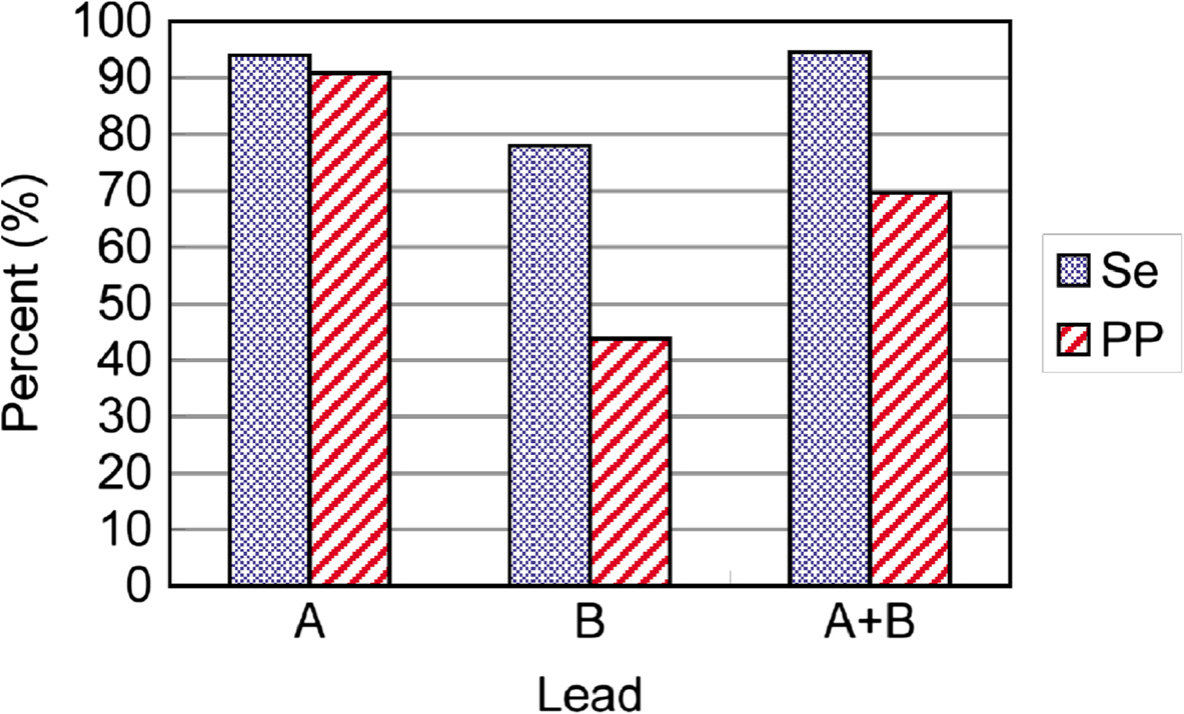
Sensitivity (Se) and positive predictive value (PP) of VEB under different lead configurations in the testing set DS2.

We investigated the effect of different lead configurations on VEB detection performance. Table 4 shows the VEB sensitivity and positive predictive value for different lead configurations on the testing set DS2, and the histogram is shown in Figure 8. These results were achieved with the optimal parameter values found in the training process. At configuration lead A, the VEB sensitivity was 93.9%, which was slightly lower than that at configuration lead A + B. However, at configuration lead A, the positive predictive value of VEB was highest at 90.9%, which was about 21% higher than that at configuration lead A + B. Therefore, from the perspective of sensitivity and positive predictive value, the detection performance of VEB at configuration lead A was the best. This was consistent with the evaluation results of the training dataset in Table 3. It also indicates that, at configuration lead A, the VEB waveform is more different from other classes of heartbeats than at other lead configurations.

**Table 4:** VEB classification performance comparison of different lead configurations under optimal parameters on testing set DS2

**Methods**	**Parameters**		**N (%)**		**V (VEB) (%)**		**Acc (%)**	**Ave (%)**
	***δ***	***C***	**Se**	**PP**	**Se**	**PP**		
Lead A	1.3	1	99.2	95.2	93.9	90.9	94.6	94.8
Lead B	0.4	1	91.4	94.6	78.1	43.8	86.5	77.0
Lead A + B	1.3	1	96.6	95.3	94.5	69.7	92.4	89.0

Ave is the average value of sensitivity and positive predictive value of class N heartbeat and VEB.

### Threshold selection in SVEB detection on the training set

**Figure 9:**
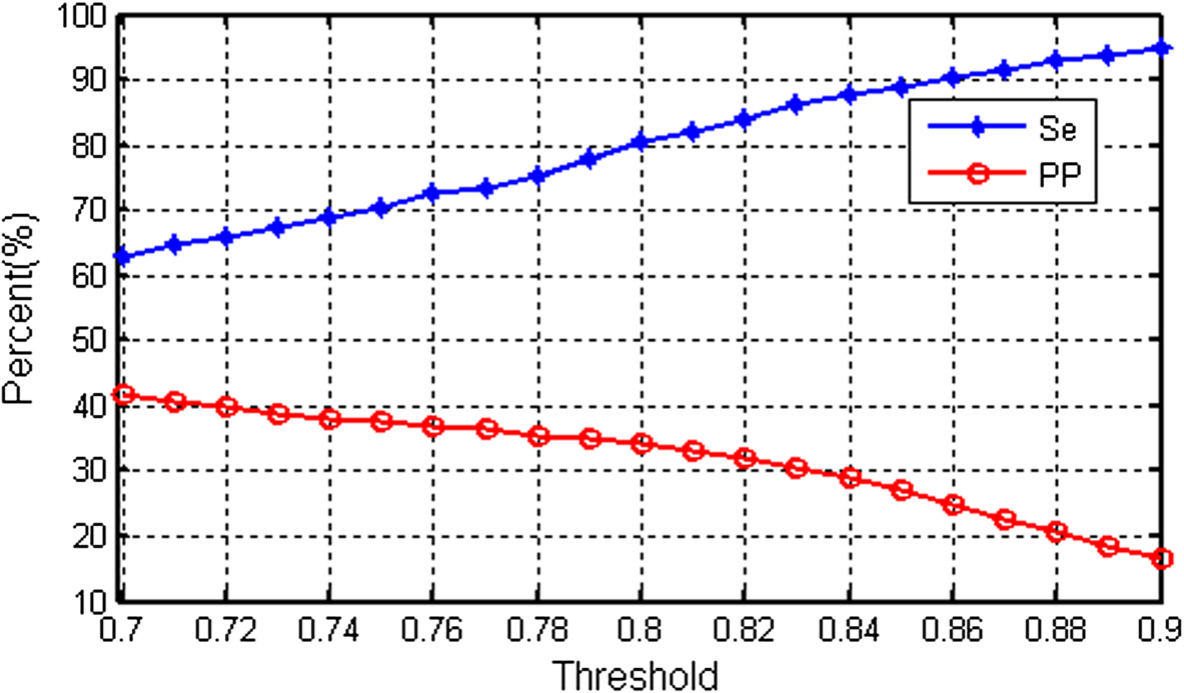
Sensitivity (Se) and positive predictive value (PP) of SVEB under different thresholds in the training set DS1.

When determining the threshold for the RR interval ratio in SVEB detection, both SVEB sensitivity and positive predictive value on the training set DS1 need to be taken into consideration. Figure 9 plots the SVEB sensitivity (blue curve) and positive predictive value (red curve) at different threshold values. As the threshold increased, the sensitivity also increased, but the positive predictive value decreased considerably. When the threshold was below 0.8, the sensitivity was also below 80%. At a threshold of 0.8, the sensitivity was close to 80%, and the positive predictive value was 34.3%. Therefore, the final threshold was set to 0.8.

### Final classification performance

According to the above results, configuration lead A was employed to obtain the best detection performance of VEB, where the penalty parameter was 1 and the kernel parameter was 0.3. The threshold value for SVEB detection was set to 0.8.

Using the already constructed classification models to detect VEB and SVEB in sequence, the final results were achieved. Table 5 shows the classification confusion matrix for VEB and SVEB detection on the testing set DS2. We calculated the performance indexes based on the information in Table 5. For VEB, the overall sensitivity was 93.9%, and the overall positive predictive value was 90.9%. For SVEB, the overall sensitivity was 91.1%, and the overall positive predictive value was 42.2%.

**Table 5:** Classification confusion matrix of class N beat, SVEB, and VEB on testing set DS2

**Reference label**	**Algorithm label**			**Total**
	**N**	**S (SVEB)**	**V (VEB)**	
N	41931	2146	181	44258
S	98	1673	66	1837
V	52	144	3025	3221
F	333	1	54	388
Q	4	0	3	7
Total	42418	3964	3329	49711

N, S (SVEB), V (VEB), F, and Q refer to heartbeats originating in the sinus node, supraventricular ectopic beat, ventricular ectopic beat, fusion heartbeat, and unknown beat type, respectively. Because we only detected VEB and SVEB, the algorithm label did not include F and Q.

As shown in Table 5, 3025 out of 3221 VEBs were detected. In addition, among the heartbeats classified as VEBs, only few actually belonged to other classes. Therefore, the sensitivity and the positive predictive value of VEB were relatively high. The system exhibited a relatively good performance in VEB detection. This is due to the fact that the random projections of VEBs together with RR intervals are very different from those of other classes of heartbeats. The selection of random projections and RR intervals as the features of heartbeats can enhance the detection performance of VEB.

In the testing set DS2, the 3329 heartbeats classified as VEBs were removed from a total of 49,711 heartbeats, and SVEB was detected from the remaining 46,382 heartbeats. As a result, 1673 beats of 1837 SVEBs were correctly classified, but among the 3964 SVEBs detected using the algorithm, 2146 beats were class N beats. Therefore, the SVEB sensitivity was relatively high, but its positive predictive value was relatively low. This is related to the fact that the RR interval ratio of SVEBs partially overlaps with that of class N heartbeats.

**Figure 10:**
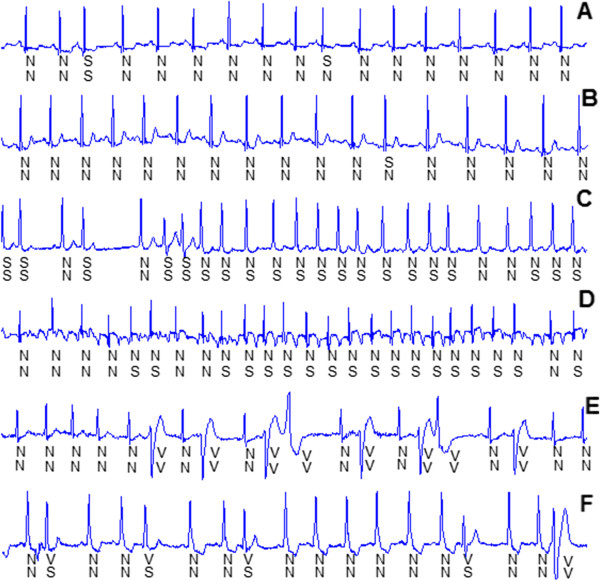
**Some SVEBs and VEBs that were classified correctly and incorrectly for six recordings. A**: One SVEB was classified correctly, and one SVEB was misclassified as class N beat for recording 100. **B**: One SVEB was misclassified as class N beat for recording 103. **C**: The previous five SVEBs were classified correctly, and the following class N beats were misclassified as SVEBs for recording 202. **D**: Many class N beats were misclassified as SVEBs for recording 222. **E**: Eight VEBs were classified correctly for recording 200. **F**: The previous four VEBs were misclassified as SVEBs and only one VEB was classified correctly for recording 214.

Table 6 shows the classification results on each recording of the testing dataset in accordance with the AAMI recommendations [15]. In the table, the accuracy for each recording was calculated based on the total number of heartbeats in that recording, and this counted in the numbers of class F and class Q heartbeats. The classification accuracy for recording 202 was 66.2%, which was the lowest among all 22 recordings. The next lowest accuracies were observed in recordings 222, 219, 231, and 213. For all the remaining recordings, the accuracy was above 95%. The SVEB sensitivities and positive predictive values for recordings 103, 219, and 231 were all zero, yet with accuracies of 99.4%, 83.2%, and 84.1%, respectively. Therefore, for each recording, the sensitivity and positive predictive value should be examined, in addition to accuracy.We will discuss a number of examples in order to explain the results. The SVEB sensitivity and positive predictive value for recording 100 were high, because only six SVEBs with RR interval ratios above 0.8 were misclassified as class N beats and only one class N beat was misclassified as SVEB. The SVEB sensitivity and positive predictive value for recording 103 were all zero, because it had only two SVEBs, and no heartbeats were classified as SVEBs. For recordings 100 and 103, the performance of class N beats improved and thus the accuracy was especially high. The results of a section of signals from these recordings are shown in Figure 10 (A) and (B). The SVEB positive predictive value for recordings 202 and 222 was only 6.3% and 24.1%, respectively, because many class N beats were misclassified as SVEBs, which led to a decrease in sensitivity for class N beats and thus a decrease in accuracy. The results of a section of signals from these recordings are shown in Figure 10 (C) and (D). The class N beats misclassified as SVEBs had shorter RR interval ratios with accompanying atrial fibrillation. The VEB sensitivity and positive predictive value for recording 200 were high because only 35 out of the 826 VEBs were classified incorrectly and no heartbeats were misclassified as VEBs. The VEBs that were classified correctly for recording 200 are shown in Figure 10 (E). The VEB sensitivity for recording 214 was low because 72 out of the 256 VEBs were misclassified as SVEBs. The VEBs that were classified incorrectly for this recording are shown in Figure 10 (F). The waveforms of VEBs were similar to those of class N beats and had no wide QRS complexes that were the main characteristic of VEB. However, owing to a better performance of class N beat, the accuracies of recordings 202 and 222 remained high.

**Table 6:** Classification performance on each recording of DS2 using AAMI standard

**Record**	**Number of beats**			**N (%)**		**S (SVEB) (%)**		**V (VEB) (%)**		**Acc (%)**
	**N**	**S**	**V**	**Se**	**PP**	**Se**	**PP**	**Se**	**PP**	
100	2239	33	1	100.0	99.7	81.8	96.4	100.0	100.0	99.7
103	2082	2	0	99.5	99.9	0.0	0.0	-	-	99.4
105	2526	0	41	100.0	99.9	-	0.0	78.0	39.5	97.6
111	2123	0	1	99.9	100.0	-	0.0	100.0	50.0	99.9
113	1789	6	0	97.1	100.0	100.0	10.3	-	-	97.1
117	1534	1	0	99.7	100.0	100.0	20.0	-	0.0	99.7
121	1861	1	1	95.5	100.0	100.0	1.2	100.0	25.0	95.3
123	1515	0	3	98.4	100.0	-	0.0	100.0	100.0	98.4
200	1743	30	826	99.1	97.7	16.7	9.6	94.6	100.0	96.6
202	2061	55	19	65.5	99.9	98.0	6.3	94.7	69.2	66.2
210	2423	22	195	98.1	98.6	94.1	22.2	83.1	94.2	96.2
212	2748	0	0	100.0	100.0	-	-	-	0.0	99.7
213	2641	28	220	99.9	88.8	72.0	85.7	96.8	81.9	88.2
214	2002	0	256	99.9	99.9	-	0.0	71.5	93.4	96.1
219	2082	7	64	83.8	99.6	0.0	0.0	87.5	86.2	83.2
221	2031	0	396	97.3	99.9	-	0.0	99.7	100.0	97.7
222	2274	209	0	75.5	98.9	90.6	24.1	-	0.0	75.0
228	1688	3	362	99.9	99.6	33.3	7.7	95.6	100.0	99.1
231	1568	1	2	84.2	99.9	0.0	0.0	50.0	100.0	84.1
232	398	1382	0	99.7	99.2	99.8	99.9	-	0.0	97.7
233	2230	7	831	100.0	99.8	66.7	66.7	99.8	92.7	97.7
234	2700	50	3	100.0	99.1	52.0	96.3	100.0	100.0	99.1
Total	44258	1837	3221	99.2	95.2	91.1	42.2	93.9	90.9	93.8

## Discussion

Based on the results, we will discuss the proposed method thoroughly in this section.

### Validation of the SVM ensemble classifiers based on the independent testing set

The aim of this study was to propose an inter-patient classification method to detect VEB and SVEB in ECG recordings of unknown patients. The performance of a heartbeat classifier often decreased when used to classify data from patients other than those whose data were used to train the classifier. Therefore, to ensure a scientifically valid assessment, the classifier must be trained on the training set to construct a good classification model, and then validated based on the independent testing set. It refers that the training set and the testing set originate from different patients. In this way, the results based on the independent testing set could reflect the actual predictive ability of the classifier.

This is an important aspect that has not been considered in a great number of studies on heartbeat classification. These intra-patient classification methods claimed to classify many heartbeat types simultaneously with high accuracy, and unfortunately, they were difficult to use in clinical applications. Patient-adaptive heartbeat classification uses local training sets from the beginning section of each test recording, and thus improves the classification performance on the rest of the recording. Despite good results, it requires expert annotation and cannot be fully automated.

To construct effective classifiers, we utilized the training set to train the SVM ensemble classifiers and to identify the optimum parameters. The same optimum values of the penalty parameter *C* and the kernel parameter *δ* for 15 SVMs were determined by leave-one-out cross-validation. In particular, the training set was divided into 22 folds with each fold including one recording, as well as different recordings originating from different patients. This contributed to the enhancement of the predictive ability of the constructed classifiers on the training set.

As shown in Table 2, the Ave (the average value of sensitivity and positive predictive value of class N heartbeat and VEB) was utilized to estimate the performance of the SVM ensemble classifiers under different parameters and lead configurations. The group of parameters with the largest Ave value was selected as the optimum parameter group, shown in Table 3. Using the optimum parameters determined on the training set, the ensemble classifiers were validated using the testing set from patients other than those who provided data used to train the classifier. This is shown in Table 4. Therefore, the results of VEB detection in Table 4 provided a valid assessment of the predictive capability for the heartbeat types in ECG recordings of unknown patients.

### The reason for using random projection and SVM ensemble to detect VEB

We proposed to use random projection to extract the morphological features of the heartbeats, which was not used in previous methods. The experimental results show that using a random matrix to calculate the random projection of a heartbeat is a very effective means of detecting VEB. This can be explained by the theory of compressive sensing. Compressive sensing has become a research hotspot in recent years. It addresses the issue of how to recover the original signal from a few measurements accurately. Its one of the central issues is the construction of a sensing matrix. Studies have demonstrated that because random matrices show very few correlations with many types of fixed transformation basis, using a random matrix as a sensing matrix can allow the system to retain information from the original signal, thereby facilitating accurate recovery of that original signal. These random matrices include the Gaussian random matrix [26]. Using a random matrix to extract features is equivalent to using a sensing matrix to measure original signals. Therefore, in the proposed method, low-dimensional random projection was used to retain the morphological information of the preprocessed heartbeats. Feature extraction is also performed alongside dimension reduction.

However, because random matrices are different each time they are generated, the extracted random features are not stable. With classifier ensembles, this instability can be used to improve the robustness of heartbeat classification. Ensemble learning techniques are commonly used in pattern classification, and they have also been applied in heartbeat classification [29-32]. To achieve satisfactory ensemble classification, the component classifiers used need to perform well and vary a great deal [33]. In the present study, SVMs, which perform better than other classifiers, were used, and the variation among classifiers was introduced using random matrices. In the final step, a simple majority vote strategy was used to determine the class of each tested heartbeat.

### Comparison with other methods

The VEB and SVEB classification results obtained in the present study were compared to those reported in previous works [1,11,19-24], as illustrated in Table 7. These methods consistently used the same database and inter-patient dataset division for testing, where the latter was defined in [1]. Table 8 lists the features extraction, feature selection, and classification methods used in previous works and the present study. As shown in Table 7, the proposed method exhibited higher VEB sensitivity and positive predictive value than methods proposed in previous studies. Its SVEB sensitivity was only slightly lower than that of one previous study [22]. Its SVEB positive predictive value was the highest of any method examined. Meanwhile, the class N heartbeat sensitivity and positive predictive value of the proposed method were not notably lower compared to other studies. The study in [22] did not provide the positive predictive values, which could lead to bias in performance assessment. This is because very low positive predictive values can affect the performance of the classification method. Maybe, the study in [22] lowered the positive predictive value to obtain high sensitivity of SVEB. Overall, the results of the comparison show that the proposed hierarchical method for heartbeat classification constitutes a substantial improvement in classification performance and is more effective in the classification of heartbeats than methods proposed in previous studies.

**Table 7:** Classification performance comparison of the proposed method with other methods following AAMI standard

**Method**	**N (%)**		**S (SVEB) (%)**		**V (VEB) (%)**	
	**Se**	**PP**	**Se**	**PP**	**Se**	**PP**
De Chazal [1]	86.9	99.2	75.9	38.5	77.7	81.9
Llamedo [11]	77.6	99.5	76.5	41.3	82.9	88.0
Mar [19]	89.6	99.1	83.2	33.5	86.8	75.9
de Lannoy [20]	80.0	-	88.1	-	78.5	-
Doquire [21]	75.9	-	82.6	-	85.1	-
de Lannoy [22]	79.8	-	92.6	-	85.1	-
Zhang [23]	88.9	99.0	79.1	36.0	85.5	92.8
Park [24]	86.3	-	82.6	-	80.9	-
Proposed method	99.2	95.2	91.1	42.2	93.9	90.9

These methods all used the same database and inter-patient dataset division for testing, where the latter is as defined in [1].

**Table 8:** Feature extraction, feature selection, and classification methods in previous works and the proposed method

**Method**	**Features**	**Feature selection**	**Classifier**
De Chazal [1]	Morphology, intervals, lead A + B	Feature set wrapper	wLDA
Llamedo [11]	WT, intervals, lead A + B	Wrapper	wLDA
Mar [19]	Morphology, WT, intervals, lead A + B	Wrapper, wLDA	MLP
de Lannoy [20]	Intervals, HOS, lead A + B	Feature set wrapper	wSVM
Doquire [21]	Morphology, intervals, HOS, lead A	Ranking	wSVM
de Lannoy [22]	Morphology, intervals, HOS, lead A	Ranking, wrapper	wCRF + L1
Zhang [23]	Morphology, intervals, lead A + B	Ranking, wrapper	SVM ensemble
Park [24]	HBF, intervals, lead A	-	Hierarchical SVM
Proposed method	Random projections, intervals, lead A	-	SVM + threshold

It is very important to extract the most effective features from the heartbeats because this has a large impact on the design and performance of the classifiers. The previous methods listed in Table 7 all used interval features and some methods also used morphology features [1,19,21-23], wavelet transforms (WT) [11,19], Hermite basis functions (HBF) [24], and high-order statistics (HOS) [20-22]. With the exception of one study [24], all previous works used feature selection techniques to choose the appropriate features from a variety of features mentioned above to improve the performance. The wLDA, wSVM, and wCRF were adopted to solve the imbalanced classification problem. However, they all used the same features to classify SVEB and VEB at a time, except for the study in [23]. Obviously, the characteristics of VEB are different from those of SVEB. The main heartbeat of VEB is a premature ventricular contraction, which has a wide waveform different from that of a normal beat; the main heartbeat of SVEB is a premature atrial contraction, which has a waveform very similar to that of a normal beat but a relatively short RR interval. Therefore, using the same features to classify these two classes of heartbeats would inevitably affect the classification performance. Zhang et al. [23] selected effective feature subsets between two classes of heartbeats to increase the performance of heartbeat classification and yet the sensitivities of SVEB and VEB were lower than the results in [19]. In another previous study [24], first Hermite basis function and three interval features were used to detect VEB, and then the interval features were used to detect SVEB. SVM was used for VEB and SVEB classifications respectively. However, since the class separability of the features used was not very strong, the classification method did not perform well enough.

In the present study, VEB and SVEB were classified using different features and classification methods. First random matrices and SVM ensemble were used to detect VEB and then the RR interval ratio was compared to a predetermined threshold to detect SVEB. In this way, the performance of the classification system was notably improved.

The proposed method in fact combines branch decision logic and artificial intelligence methods and shows the advantages of both methods. The branch decision logic method is simple and fast, and the judgment rule can be included. Using the artificial intelligence method, the classification model identifying different classes of heartbeats can be learned from a dataset. Then, unknown heartbeats were classified using this learned model.

In addition, some studies only reported SVEB and VEB sensitivities but did not report their positive predictive values [20-22,24]. Oftentimes, although the sensitivity of certain heartbeat classes is high, due to the fact that the heartbeats detected include many heartbeats that are actually of other classes, the positive predictive value is relatively low. In this case, the performance of the classification system is still not satisfactory. The SVEB positive predictive value is relatively low for all methods, always below 50%. The SVEB detection is more challenging than the detection of VEBs. Features that can be used to identify SVEB effectively still need to be explored.

### Classification time required by the system

Table 9 shows the time needed for identifying VEB and SVEB using the proposed method. Training time is not counted here. The hierarchical heartbeat classification system we proposed not only showed satisfactory classification performance in the detection of VEB and SVEB but also ran relatively fast. As shown in Table 9, not including detection time of the QRS complex, time used to identify VEBs from the 49,711 heartbeats in the testing set DS2 using random projections and SVM ensemble was 832 s. Of this time, 4.4 s was used to calculate the random projections. Time used to identify SVEBs from the remaining heartbeats was 0.1 s. The total classification time was about 832.1 s, less than 14 minutes. Analysis of each heartbeat took 0.02 s, equivalent to analyzing about 3000 heartbeats per minute. The time required to calculate the random projections and SVM ensemble for VEB detection was shorter, and the speed of SVEB detection using threshold was much faster compared to other methods. The proposed system does not compromise classification time because it uses a hierarchical strategy.

**Table 9:** Time of detecting VEB and SVEB

**Heartbeat class**	**Classification time(s)**	**Classification time per beat(s)**
VEB	832.0	0.02
SVEB	0.1	0.000002
Total	832.1	0.02

The values in the table were only kept one significant digit after the decimal point.

### Application of the proposed hierarchical method

Heartbeat classification has been studied for decades. However, the results of heartbeat classification on clinical data are not satisfactory. The inter-patient classification method is constructed based on independent training and testing sets and thus it has a good predictive ability. However, only a few studies have been conducted. Owing to the difficulty of inter-patient heartbeat classification, a high performance classification cannot be obtained when a number of heartbeat types are classified simultaneously. The AAMI-recommended practice provides a protocol for a reproducible test with realistic clinical requirements. This was utilized to assess the performance of different methods [15]. The AAMI recommends five classes, including N, S (or SVEB), V (or VEB), F, and Q. SVEB and VEB are important from a clinical perspective, and can indicate risks for patients with cardiovascular diseases. The class N beat, SVEB, and VEB include five, four, and two heartbeat types, respectively [1]. Thus, together they contain eleven heartbeat types and account for 99.2% of all the heartbeats in the MIT-BIH arrhythmia database, with the exception of four paced recordings (102,104,107, and 217). Furthermore, other heartbeat types in the class N beat, SVEB and VEB can be detected. Therefore, the detection of SVEB and VEB from class N beats can address the clinical concerns, and it is not limited in terms of heartbeat classification. Because present methods cannot provide satisfactory detection results of SVEB and VEB, we proposed a hierarchical method that adopted different features and classification methods for VEB and SVEB detection.

To perform a fully automatic heartbeat classification using the proposed method, automatic identification of the QRS complex is required. The QRS detection module can be performed prior to the preprocessing of heartbeats. Many methods are available for QRS detection with accuracy rates greater than 99.5% [34-36].

The proposed method can be ported to a real-life heartbeat classification system to detect VEB and SVEB with some fine-tuning. The MIT-BIH arrhythmia database has a sample frequency of 360 Hz. The ECG signals with a different sample frequency and amplitude maximum need to be processed to remove the signal variation due to the sample frequency and amplitude. To obtain an improved classification performance, the users can re-train our proposed classification model using their own data prior to predicting the heartbeat types of other unknown patients. In addition, the proposed method can be utilized for teaching purposes in order to help students to understand the work process of medical pattern recognition.

The core idea of the proposed method is that different features and classification methods were adopted to detect different heartbeat types in order. Although the proposed method was used for the detection of VEB and SVEB, class F beat and a few heartbeat types in a class could be detected in terms of the core idea of the method. In addition to this, the random projection and SVM ensemble classification for VEB detection could be extended to other medical data to improve the classification performance.

## Conclusions

A hierarchical automated heartbeat classification system based on the inter-patient data partitioning and AAMI guidelines was proposed. Different features and classification methods were used to identify VEBs and SVEBs separately. Low-dimensional random projections retained the information of the preprocessed heartbeats, achieving feature extraction and dimension reduction at the same time. The randomness of random matrices was utilized to construct SVM ensemble classifiers and improve VEB detection performance. The RR interval ratio was used to further distinguish SVEBs from class N heartbeats. This was achieved using a simple comparison between the RR interval ratio and a predetermined threshold. The classification models were all constructed on the training dataset, and the obtained optimal configuration was used in the independent testing dataset to assess the final performance of the classification system. The proposed method here achieved significantly better classification than previous methods. In addition, the running speed of this new method is also fast, and analysis of each heartbeat only takes 0.02 s on average. The proposed method in fact combines the advantages of the branch decision logic method and those of the artificial intelligence method. It not only learns the classification model identifying different classes of heartbeats from the data but also allows inclusion of a judgment rule.

## Abbreviations

AAMI: Association for the Advancement of Medical Instrumentation; VEB or V: Ventricular ectopic beat; SVEB or S: Supraventricular ectopic beat; SVM: Support vector machine; ECG: Electrocardiogram; N: Heartbeat originating in the sinus node; F: Fusion heartbeat; Q: Unknown beat type; wLDA: Weighted linear discriminant analysis; wSVM: Weighted support vector machine; wCRF: Weighted conditional random field; RBF: Radial basis function; Se: Sensitivity; PP: Positive predictive value; Acc: Accuracy; Ave: Average value of sensitivity and positive predictive value; WT: Wavelet transform features; HBF: Hermite basis functions; HOS: High-order statistical characteristics.

## Competing interests

The authors declare that they have no competing interests.

## Authors’ contributions

HH and GH conceived of the study and designed the experiments. HH performed the experiments. All authors participated in the analysis of data. HH and GH wrote the manuscript. All authors read and approved the final manuscript.

## Authors’ details

HH: She is currently a lecturer at Department of Biomedical Engineering, Beijing Jiaotong University, Beijing, China. She got her Master degree and PhD degree from the Department of Biomedical Engineering, School of Medicine, Tsinghua University, Beijing, China. She has about over 10 years of research experience in Medical Pattern Recognition, including feature extraction and classifier design. She has performed study about iris recognition and ECG heartbeat classification. Now she conducts research work about the psychiatric disorder multivariate pattern classification based on fMRI, such as ADHD and depression. JL: He is a Professor in the Department of Biomedical Engineering, Beijing Jiaotong University, Beijing, China. His research interests are Biomedical Signal/Image Processing and Medical Imaging. QZ: She is an Associate Professor in the Department of Biomedical Engineering, Beijing Jiaotong University, Beijing, China. Her research interests are Biomedical Signal Processing. RW: She is an Associate Professor in the Department of Biomedical Engineering, Beijing Jiaotong University, Beijing, China. Her research interests are Biomedical Signa/Image Processing and Medical Pattern Recognition. GH: He is a Professor in the Department of Biomedical Engineering, School of Medicine, Tsinghua University, Beijing, China. His research interests are Biomedical Signal/Image Processing, Medical Pattern Recognition and Digital Medical Instrumentation. His research work is focused on the detection and processing of ECG, EEG and evoked potential signals, X-ray image cardio-cerebral angiography, CT and MRI images, iris images, multifocal electro-oculography feature extraction and clinical application, the application of DSP in clinical instruments etc.
